# Giant Cell Tumor of the Patellar Tendon Sheath: A Rare Case of Anterior Knee Pain

**DOI:** 10.7759/cureus.1690

**Published:** 2017-09-16

**Authors:** Ömer Faruk Kılıçaslan, Yusuf alper Katı, Ozkan Kose, Bekir Erol, Arsenal Sezgin Alikanoglu

**Affiliations:** 1 Department of Orthopaedics and Traumatology, University of Health Sciences, Medical Faculty, Antalya Education and Research Hospital, Antalya, Turkey; 2 Department of Radiology, University of Health Sciences, Medical Faculty, Antalya Education and Research Hospital, Antalya, Turkey; 3 Department of Pathology, University of Health Sciences, Medical Faculty, Antalya Education and Research Hospital, Antalya, Turkey

**Keywords:** giant cell tumor, patellar tendon, nodular synovitis, knee

## Abstract

Giant cell tumor of the tendon sheath (GCTTS) is a benign, proliferative lesion of the synovium of the joint, the bursa, and the tendon sheath. We report a case of intra-articular, localized GCTTS arising from the patellar tendon, which is a rare cause of anterior knee pain. The diagnosis may be delayed due to non-specific symptoms and normal plain radiographic findings. Magnetic resonance imaging (MRI) is diagnostic to detect the lesion, but several other clinical entities and lesions should be evaluated in the differential diagnosis. The treatment of choice is a total excision of the lesion without leaving residual tumor tissue, in order to reduce the risk of recurrence. We present a patient with GCTTS of the patellar tendon and discuss its clinical and radiographic characteristics, differential diagnosis, pathology, and treatment.

## Introduction

Giant cell tumor of the tendon sheath (GCTTS) is a benign, proliferative lesion of the synovium of the joint, bursa and tendon sheath, which was first described by Jaffe, et al., in 1941 [1]. Although histologically identical, it has two distinct forms according to its pattern of involvement and biologic behavior, namely, diffuse and localized. Local forms are confined to a distinct area of synovium or tendon sheath, whereas diffuse forms demonstrate extensive involvement of the whole synovial membrane and capsule [2]. Local GCTTS usually involves flexor tendons of the hand and foot and extra-articular involvement. Diffuse form, also called pigmented villonodular synovitis (PVNS), is usually intra-articular and involves large synovial joints, such as the knee, ankle, and shoulder. The knee joint is usually involved in a diffuse pattern (PVNS), whereas intra-articular GCTTS of the patellar tendon is a rare lesion [3]. We present a case of GCTTS involving the infrapatellar fat pad and discuss its clinical and radiographic characteristics, differential diagnosis, pathology, and treatment.

## Case presentation

**Figure 1:**
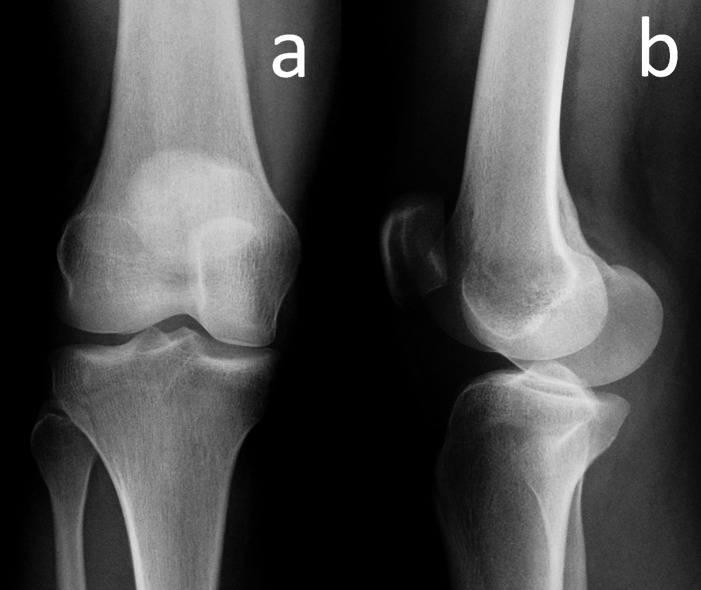
Anteroposterior and lateral knee radiographs

A 32-year-old woman presented to our outpatient clinic with the complaints of right anterior knee pain that had been gradually increasing in severity over the past six months. Recently, she noticed a herniating tumoral mass at the joint line, medial to the patellar tendon, particularly prominent in flexion of the knee. She was a housewife, had no history of previous trauma to her knee, and had not experienced clicking, locking, or giving way. Her past medical history revealed no abnormality. On physical examination, there was a palpable, mobile mass, medial to the patellar tendon. The knee movements were in the normal range without pain. There were no signs of meniscal pathology or ligamentous instability, and patellar movements were normal. The neurovascular examination was also normal. Plain anteroposterior and lateral radiographs revealed no bony abnormality of the right knee joint (Figure 1).

**Figure 2:**
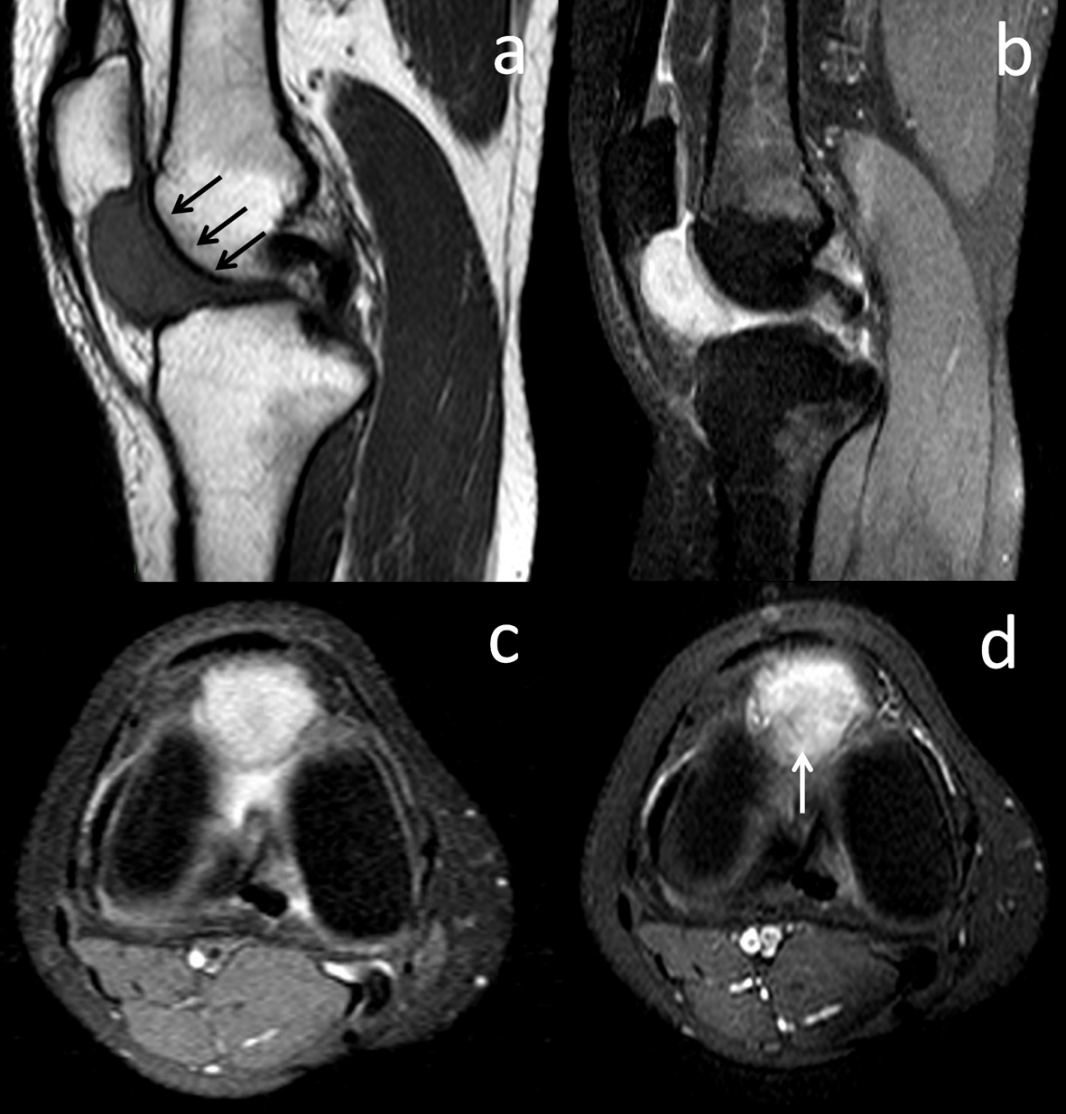
Preoperative MRI examination of the knee. MRI: magnetic resonance imaging

Magnetic resonance imaging (MRI) of the knee joint revealed an encapsulated, well-localized soft tissue mass in the fat pad deep to the patellar tendon. A sagittal turbo spin-echo T1-weighted MRI showed an ovoid mass (arrows) with a slightly hyperintense signal relative to the skeletal muscle occupying the anterior joint space of the knee (Figure 2a). Sagittal and axial fat suppressed proton density; the MRI showed a heterogeneous, high-signal intensity in the mass (Figure 2b, 2c). Contrast-enhanced T1-weighted spectral presaturation with inversion-recovery axial MRI showed enhancement of lesion. Hypointense foci belonging to hemosiderin deposits were present at the center of the lesion (Figure 2d).

**Figure 3:**
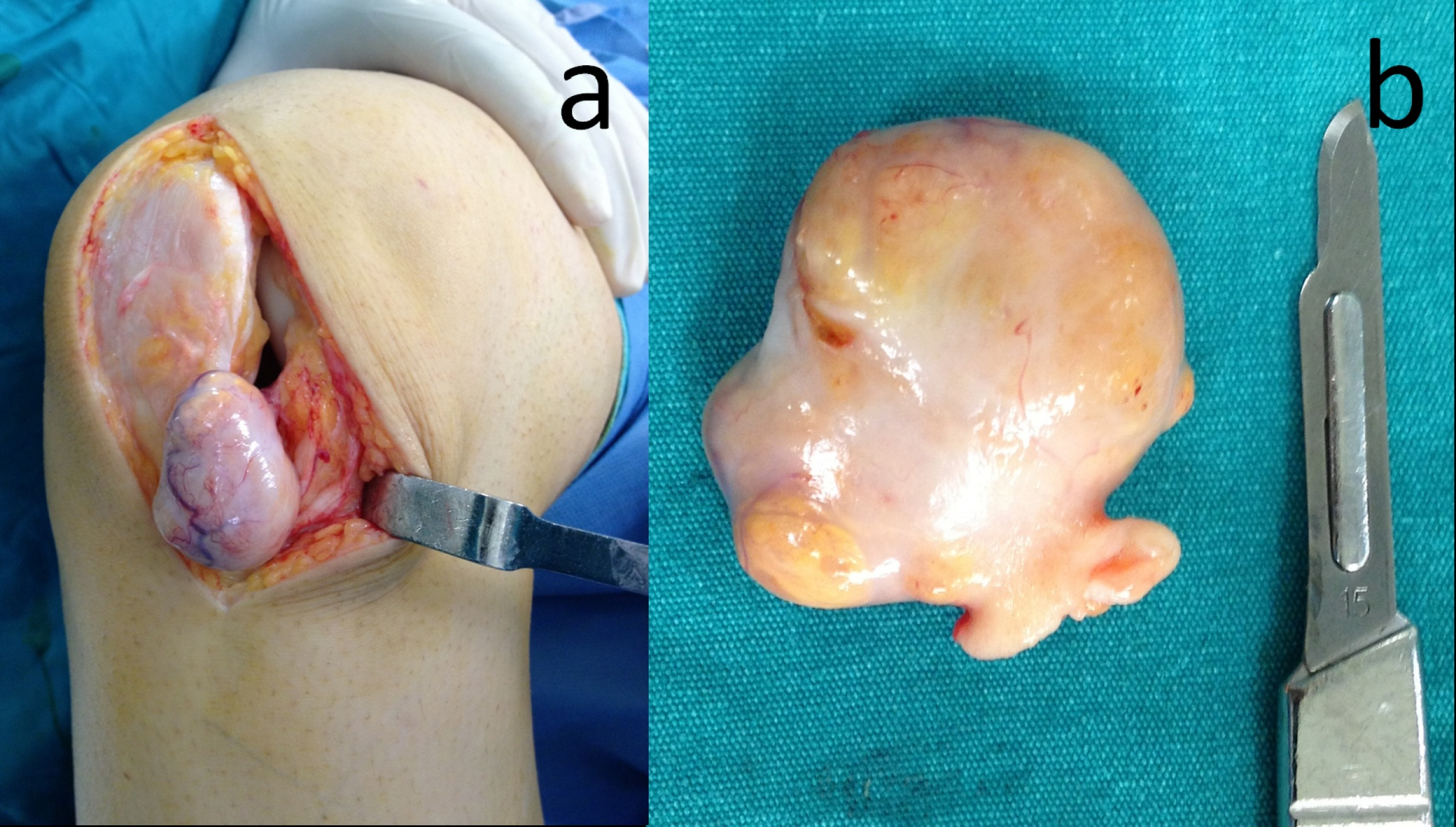
Intraoperative appearance of the lesion.

The patient underwent an ultrasound-guided, fine needle aspiration biopsy. The biopsy was reported as a benign fibrohistiocytic lesion. The differential diagnoses of an intraarticular mass located in the fat pad includes GCTTS (nodular synovitis), Hoffa’s disease, chondroma or osteochondroma of the infrapatellar fat pad, synovial sarcoma, malignant fibrous histiocytoma, intra-articular lipoma, ganglion cyst, parameniscal cyst arising from anterior horn of the menisci, gout tophus, and arthrofibrosis secondary to previous knee surgery. The patient underwent total surgical excision of the mass under spinal anesthesia with medial knee arthrotomy (Figure 3a). The tumor was a yellowish-gray color, capsulated, pedunculated, 35 x 30 x 18 mm in size, and rubbery in consistency (Figure 3b). 

**Figure 4:**
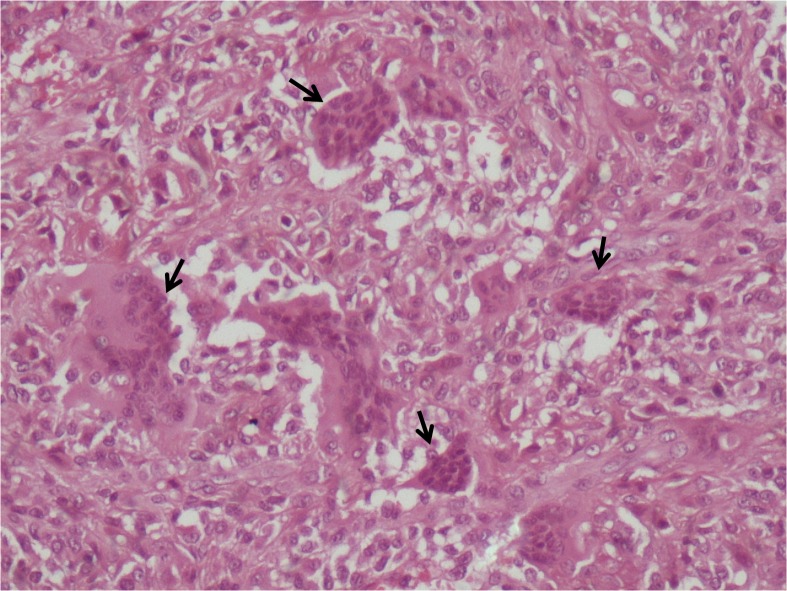
Histopathological examination shows the multinucleated giant cells (H&E, X400) H&E: haemotoxylin and eosin stain

Histologically, the lesion was composed of fibrous tissue containing fibroblasts, histiocytes, and foamy macrophages, as well as scattered multinucleated giant cells. No mitotic figures, nuclear atypia, or necrosis were noted (Figure 4). The lesion was diagnosed as a giant cell tumor of the tendon sheath, based on both radiological and histological findings. 

**Figure 5:**
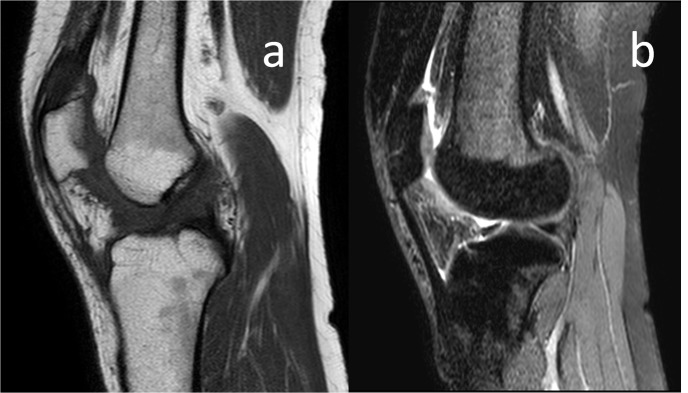
Final control MRI examination of the patient shows no signs of recurrence. MRI: magnetic resonance imaging

The postoperative period was uneventful, and the patient started weight-bearing on the second postoperative day. Knee range of motion exercises were encouraged within the first week. At the third month follow-up after the surgery, she had complete relief of symptoms without evidence of recurrence. The patient returned to her previous level of activity with a normal gait and full range of knee motion. At the final follow-up, 18 months after the surgery, she was still free of symptoms and the final control MRI was evaluated as normal without evidence of recurrence (Figure 5).

## Discussion

Localized, intra-articular GCTTS of the knee joint is a rare lesion and a rare cause of anterior knee pain. In a retrospective study on 207 GCTTS cases, only eight cases of knee involvement have been reported [4]. GCTTS affects individuals between the ages of 30-50 years and is more often seen in women than in men. The extra-articular GCTTS usually presents with an increasing painless mass in the extremities, especially on digits of the hand and foot [1, 5]. However, its intra-articular counterpart may remain asymptomatic until it becomes large enough to cause mechanical symptoms and its prominence can be noticed by the patient. There is no specific symptom for GCTTS of the patellar tendon, but joint effusion, painless and/or painful mass, swelling, tenderness, locking, limitation of knee movements have all been reported [5-6].

Imaging studies play an important role in the diagnosis. Conventional radiography is used as the first-step diagnostic tool for the evaluation of patients with knee pain. In most cases, radiographs are normal [5, 7]. Sometimes, areas of soft tissue opacities may replace the Hoffa fat pad lucency [8]. Coexisting joint effusions may be inspected on radiograms. Some studies found that extrinsic erosion of bone was found in up to 20% of patients [7]. Magnetic resonance (MR) examination is highly sensitive for identification and diagnosis of these lesions. On MRI, the lesion is seen as an oval, solitary mass confined to the knee joint. MR imaging findings are variable. The typical appearance is composed of a well marginated, ovoid mass or mass with lobulated contours. It is intermediate or slightly hyperintense on T1-weighted images relative to skeletal muscle, whereas on T2-weighted images it shows variable signal intensity. It contains low signal intensity regions due to the hemosiderin deposits which are much more apparent on gradient echo images. Differential diagnosis includes several pathologic processes. It can be differentiated from the PVNS due to the lack of frond-like projections of synovium, which is a characteristic feature of PVNS. Also, there is a lack of constriction effect of the PVNS surrounding joint, unlike pedunculated growth of localized nodular synovitis. Inflammation and fibrosis of the infrapatellar fat pad, namely Hoffa’s disease, differs from localized intra-articular GCTTS because of the indeterminate margins seen on MRI scans. Chondroma or osteochondroma of the infrapatellar fat pad has the same signal characteristics as cartilage or bone marrow. In post-surgery, fibrosis strands of low signal intensity and the presence of operation materials reveal the diagnosis. Lipomas have characteristic MRI features proven by fat suppression sequences. Parameniscal cysts are accompanied by meniscal tears. Sometimes, linear collection of fluid due to the shear injury may resemble the entity, but secondary signs of anterior cruciate ligament (ACL) injury are usually evident [9]. Although MR appearance of tophaceous gout is nonspecific, clinical history, laboratory values, and the absence of hemosiderin deposits may be helpful for exclusion [10]. Ganglion cysts typically present high-signal on T2 and short tau inversion recovery (STIR) images, and they do not enhance with contrast media (that, or they do not contain hemosiderin) [9]. As for the soft tissue sarcomas, some specific signs like triple sign and bowl of grapes sign, together with soft tissue calcifications, and poorly defined margins suggest the diagnosis.

However, a definitive diagnosis can be made with pathological examination. Histologically, it consists of fibrous tissue (collagenous stroma) that contains pleomorphic cell population, including lipid-laden foam cells, multinucleated giant cells, and round or polygonal stromal cells, often with deposits of haemosiderin. Mild nuclear pleomorphism is often seen, but necrosis is rare [2].

The standard treatment for GCTTS is total excision of the tumor. The most common complication seen after surgery is the local recurrence of the lesion. When the tumor is localized, total excision is usually curative with a low rate of recurrence. However, in case of diffuse involvement (PVNS of the knee), it is hard to completely remove the lesion with surgery because all synovial linings of the joint space are affected. Residual tumors are the main reason for recurrence. In diffuse form, four-portal arthroscopy, the posterior compartment of the knee joint should be included and care taken to not leave any residual tumor. Adjuvant treatments, such as intra-articular radioactive isotope (90-Yttrium) injection or external beam radiotherapy, can be used to decrease the risk of recurrence [2]. In our case, the lesion was localized, and we preferred open surgery to remove the lesion without leaving any residual tissue. On the other hand, arthroscopic removal of localized GCTTS of the knee has also been reported [6].

## Conclusions

In conclusion, GCTTS may involve the patellar tendon and cause subtle clinical signs and symptoms during the initial stage of the disease when the tumor is still small in size. Any prominence around the patellar tendon associated with pain should alert the physician of a possible intra-articular tumoral mass. Because the radiographs are usually normal, further advanced imaging techniques, such as MRI, should be ordered in case of suspicion. Total excision of the tumor results in a good prognosis, although recurrence may occur.
